# On the effectiveness of proton boron fusion therapy (PBFT) at cellular level

**DOI:** 10.1038/s41598-022-23077-0

**Published:** 2022-10-27

**Authors:** Mehrdad Shahmohammadi Beni, M. Rafiqul Islam, Kyeong Min Kim, Dragana Krstic, Dragoslav Nikezic, Kwan Ngok Yu, Hiroshi Watabe

**Affiliations:** 1 Department of Physics, City University of Hong Kong, Tat Chee Avenue, Kowloon Tong, Hong Kong, China; 2 Division of Radiation Protection and Safety Control, Cyclotron and Radioisotope Center, Tohoku University, 6-3 Aoba, Aramaki, Aoba-Ku, Sendai, Miyagi 980-8578 Japan; 3 Graduate School of Biomedical Engineering, Tohoku University, Sendai, 980-8579 Japan; 4 Korea Institute of Radiological & Medical Sciences, 75, Nowon-Ro, Nowon-Gu, Seoul, Korea; 5 Faculty of Science, University of Kragujevac, Kragujevac, Serbia; 6 State University of Novi Pazar, Novi Pazar, Serbia

**Keywords:** Surgical oncology, Radiotherapy

## Abstract

The present work introduced a framework to investigate the effectiveness of proton boron fusion therapy (PBFT) at the cellular level. The framework consisted of a cell array generator program coupled with PHITS Monte Carlo package with a dedicated terminal-based code editor that was developed in this work. The framework enabled users to model large cell arrays with normal, all boron, and random boron filled cytoplasm, to investigate the underlying mechanism of PBFT. It was found that alpha particles and neutrons could be produced in absence of boron mainly because of nuclear reaction induced by proton interaction with ^16^O, ^12^C and ^14^N nuclei. The effectiveness of PBFT is highly dependent on the incident proton energy, source size, cell array size, buffer medium thickness layer, concentration and distribution of boron in the cell array. To quantitatively assess the effectiveness of PBFT, of the total energy deposition by alpha particle for different cases were determined. The number of alpha particle hits in cell cytoplasm and nucleus for normal and 100 ppm boron were determined. The obtained results and the developed tools would be useful for future development of PBFT to objectively determine the effectiveness of this treatment modality.

## Introduction

**Figure 1:**
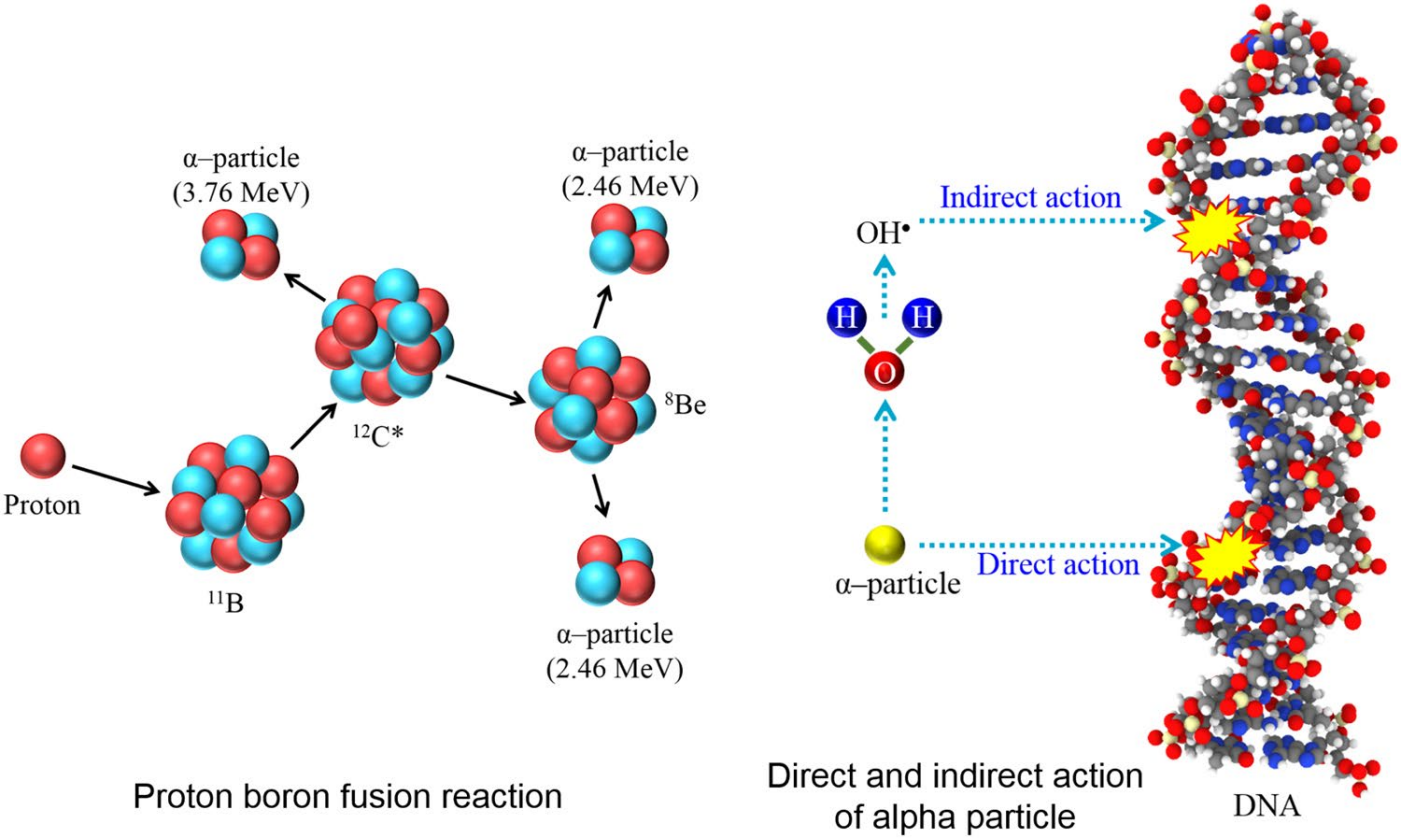
Schematic diagram showing proton boron (^11^B) fusion reaction and the alpha particle interaction with the DNA.

The ultimate goal of radiation therapy is to eradicate cancer cells while limiting damage to normal healthy cells^1–6^. Considering this, researchers have proposed enhancement techniques to the currently employed radiotherapy modes. Proton boron fusion therapy (PBFT) is one of the recently reported enhancement techniques, aiming to induce more effective damages to the cancer cells using short range and densely ionizing alpha particles^7^. Alpha particles are densely ionizing and have lower lateral scattering^8^, leading to highly clustered damages such as DNA double strand breaks^9–11^. The alpha particles generated in the PBFT have high linear energy transfer (LET) radiation and are biologically effective^12,13^, which is desirable for killing tumor cells. The mechanisms for production of alpha particles in PBFT and the subsequent direct and indirect actions of the alpha particles are shown schematically in Fig. 1.

The idea of using proton boron fusion reaction as a means of radiation therapy was first proposed by Yoon et al.^7^, with the aim to achieving cellular level treatment and enhancing the biological effectiveness of cancer treatment. Short-range alpha particles are produced in the proton boron fusion reaction (p + ^11^B → 3α). Upon the interaction of a proton with boron (^11^B), an excited state carbon (^12^C) nucleus will be formed, which decays into an alpha particle (with energy of 3.76 MeV) and a beryllium (^8^Be) nucleus. The ^8^Be nucleus can further decay into two alpha particles with energy of 2.46 MeV (see Fig. 1). In a follow-up study, the PBFT was first proven experimentally by Cirrone et al.^14^, where human prostate cancer cells DU145 were exposed to a 62 MeV proton beam at the superconducting cyclotron of the INFN-LNS facility at Catania, Italy. Cirrone et al.^14^ gave evidence that PBFT could enhance the radiation therapy and demonstrated potentials for future developments in the field of radiation therapy. In another study, the application of PBFT to proton therapy was studied and the generated alpha particles were detected^15^; this was accomplished through the employment of a medical proton accelerator (incident proton energy of 79.7 MeV) and fine-grained nuclear emulsion films for detection of alpha-particle tracks. However, recently the effectiveness of equivalent dose for PBFT for brain cancer was also studied^16^; this was performed using the MCNP (Monte Carlo N Particle) code to model the Snyder head phantom that consisted of brain, skull, skin and a tumor region. Interestingly, the authors expressed that “*from the Monte Carlo standpoint, the effectiveness of the proton boron fusion therapy is not related to the alpha particles because the dose component of alpha particles is negligible*”; which however did not agree with previously reported data.

Considering the equivocal reported results on the effectiveness of PBFT, and taking into account that radiation-induced damages to the DNA within the cell nucleus can lead to cell death, we strongly believe that it is pertinent to investigate PBFT, particularly its effectiveness and factors that influence its efficiency, in different cellular constituents (i.e., cell nucleus and cytoplasm). Crucial factors including the incident energy of the proton beam, beam size, spatial distribution of boron in cell cytoplasm and the buffer medium thickness above the cell array can affect the effectiveness of PBFT, through the produced alpha particles or other mechanisms.

In the present work, we developed an open-source terminal-based computer program for modelling large cell arrays with uniform and random boron distributions. The program was coupled with the Particle and Heavy Ion Transport code System (PHITS) Monte Carlo package (https://phits.jaea.go.jp/). The program also enabled users to generate PHITS input script for a large cell array, which simplified the error-prone task of preparing long input scripts for Monte Carlo simulation packages. Using the functions provided in this computer program would help unify the results among different researchers and in turn reduce discrepancies in the reported results. The findings in the present work and the developed software would be useful for future studies related to PBFT investigations.

## Materials and methods

### Program structure and features

**Figure 2:**
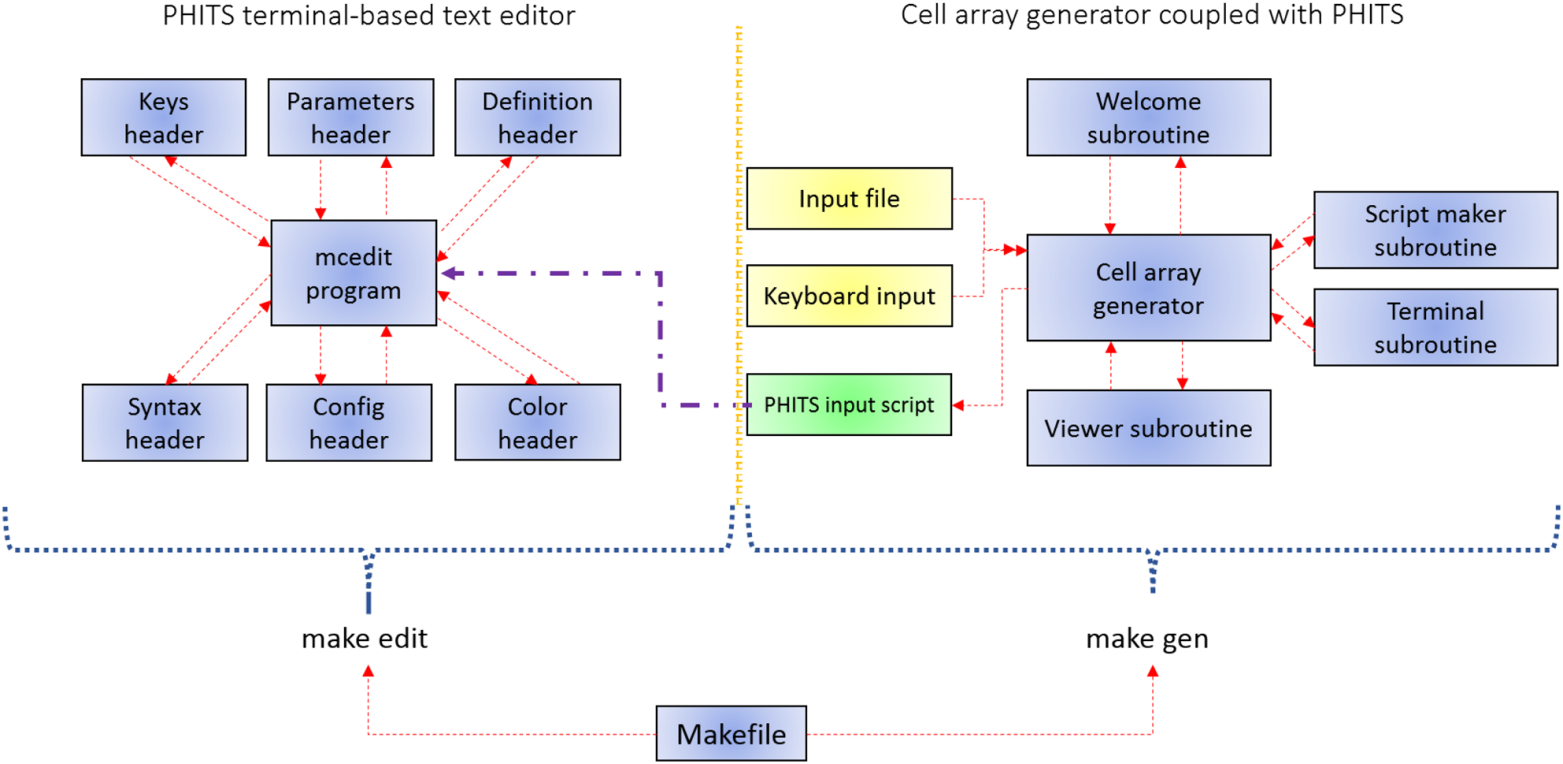
Flowchart of the cell array generator and PHITS text editor programs developed in the present work.

The main cell array generator program was developed using the FORTRAN90 programming language and has about ~ 1600 lines of code. A dedicated terminal-based text editor, named mcedit for PHITS Monte Carlo package was developed using C programming language having about ~ 1000 lines of code. The entire program was coupled with gnuplot to visualize the generated cell array structure and boron distribution in the modelled array. The program flowchart is shown in Fig. 2.

The cell array generator program generates cellular structures by taking 12 user inputs: (1) *n*, (2) *lcell*, (3) *tcell*, (4) *lnuc*, (5) *tbuffer*, (6) *airad*, (7) *maxcas*, (8) *maxbch*, (9) *e0*, (10) *sobp*, (11) *nsobp*, (12) *r0*, (13) *ppm*, (14) *cytob*, (15) *nuclb*, (16) *watb*, (17) *ppmw* and (18) *omp*. The definition of these 12 user inputs are shown in Table 1.

**Table 1:** Definition of user inputs for the cell generator program for simulation of PBFT.

User input	Definition	User input	Definition
*n*	Array size (n^2^)	*lcell*	Cell length (µm)
*tcell*	Cell thickness (µm)	*lnuc*	Nucleus length (µm)
*tbuffer*	Buffer medium thickness (µm)	*airad*	Ambient air radius (cm)
*maxcas*	History number per batch	*maxbch*	Number of batches
*e0*	Input energy (MeV)	*sobp*	SOBP beam option (0: off, 1: on)
*nsobp*	Number of SOBP points	*r0*	Beam radius (mm)
*ppm*	Concentration of boron (ppm)	*cytob*	Boron cytoplasm (0: normal, 1: boron, 2: random boron)
*nuclb*	Nucleus boron (0: off, 1: on)	*watb*	Buffer boron (0: off, 1: on)
*ppmw*	Concentration of boron in buffer medium (ppm)	*omp*	Parallelization (0: off, 1: on)

The cell generator program was coupled with gnuplot (http://www.gnuplot.info/) to facilitate visualization of the modelled cell array. This would be particularly helpful when analyzing the modelled structure and distribution of boron in cell cytoplasm. In addition to this, a quick terminal visualization was also developed to help assist users to check the boron distribution in the modelled cell array. It is important to note that the MC computations are in fact numerical tools and are an indispensable part of radiobiological experiments. The ultimate aim of the present work is to numerically investigate the effectiveness of PBFT and unfold its true nature.

### Cell array structure and boron distribution

**Figure 3:**
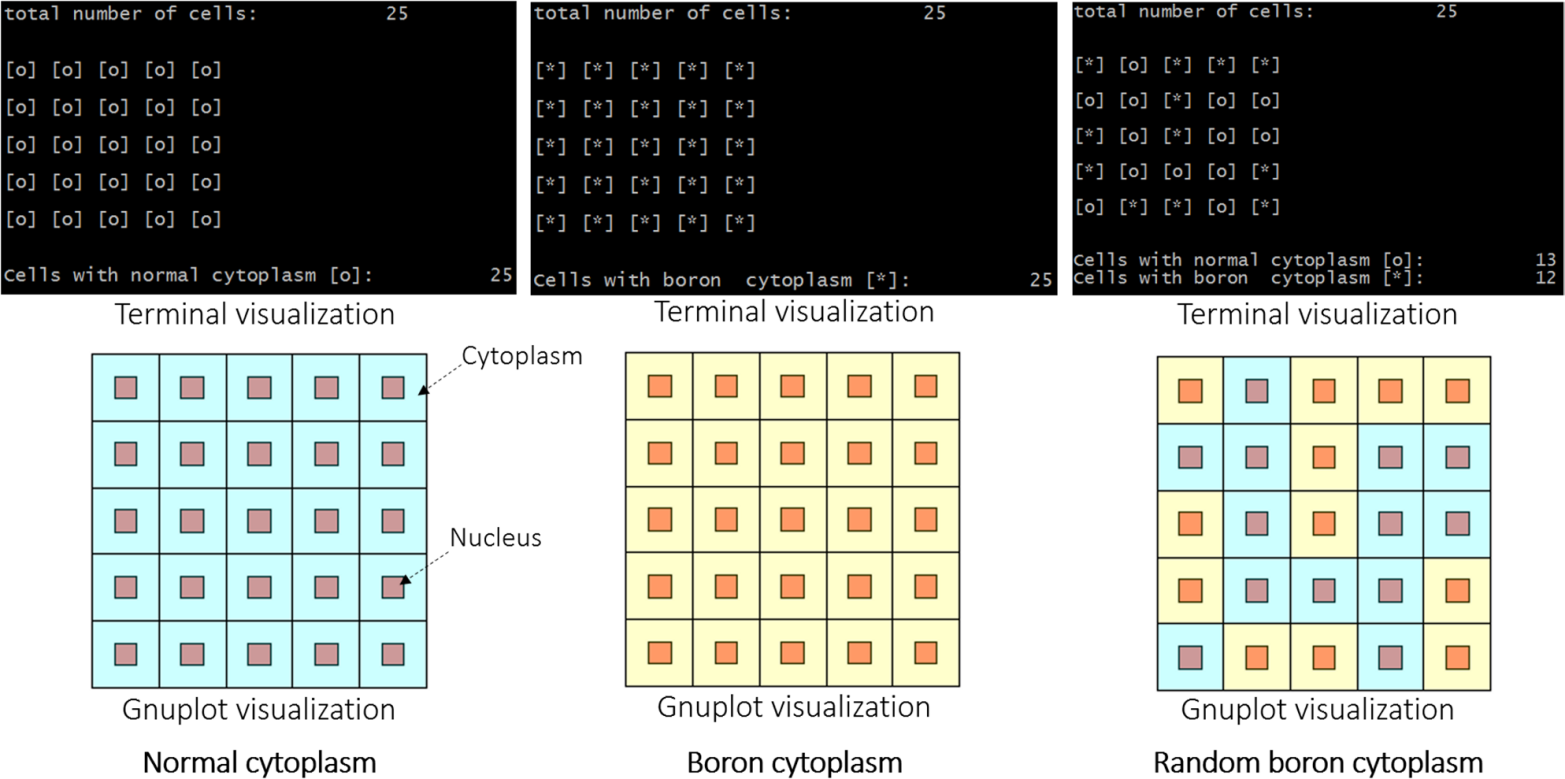
Normal, boron filled, and random boron filled cytoplasm in the modelled cell array visualized by terminal and gnuplot visualization tools embedded in our program (blue represents normal and yellow represents boron filled cytoplasm).

The cells were modelled as rectangular parallelepiped (RPP) by taking the user inputs *lcell* and *tcell*, while the nucleus of the cell was assumed to be a cuboid with length of *lnuc* placed at the center of a modelled cell. The cells were assumed to be at 100% confluence, i.e., packed next to one another without any spacing^17,18^. It is remarked that each cell has separate domains for the nucleus and cytoplasm, and boron uptake could be controlled in the cytoplasm and the nucleus. The presence of boron in the cell nucleus could be controlled by the users using *nuclb* variable shown in Table 1. In the boron filled cases shown in the present work, the boron presence in the nucleus was also considered. Three cases of boron uptake were considered, namely, (1) no boron (normal), (2) all boron and (3) random boron presence in the cytoplasm of cells in the modelled array. The boron distribution in the cell cytoplasm and nucleus was decided using uniformly distributed random numbers between 0 and 1. If the generated random number is smaller than 0.5, the cell would be considered to contain boron; else the cell would have normal cytoplasm without any boron. These were implemented in the program in form of an array that was used to check a variable named “*borcheck*”. If *borcheck* = 0, the generated cell would have no boron; if *borcheck* = 1, the cell cytoplasm and nucleus (if nucleus boron option was turned on) would contain a user-defined amount of boron in ppm. The results from a test for normal, boron filled and random boron distribution in the modelled cell array are shown in Fig. 3. These were obtained from the terminal and the coupled gnuplot visualization tool embedded in our program.

Realistically, cells have complex geometrical structure and chemical composition. It is rather tedious to consider all possible geometrical features and chemical compositions, given that different types of cells would have distinct characteristics. Previously, various investigators considered monolayer cells^19–21^, which are also considered in the present work for the modelled cell array, with the additional detail that separates cytoplasm and nucleus domains as adopted in the previous work of Clarke and Jevremovic^22^, which was also used in our previous work^23^.

### Material compositions and densities

There are three main domains in the present program, namely, cell cytoplasm, nucleus, and buffer medium above the cell array. The material composition for cell cytoplasm and nucleus were taken from our previous study^23^. The material composition and density of cell cytoplasm, nucleus and buffer medium are summarized in Table 2.

**Table 2:** Material composition (in weight %) and density of cell cytoplasm, nucleus and buffer medium.

Cell cytoplasm	Cell nucleus	Buffer medium
Density: 1.27 g/cm^3^	Density: 1.27 g/cm^3^	Density: 1.00 g/cm^3^
^1^H: 10.64%	^1^H: 10.64%	^1^H: 11.1%
^16^O: 74.5%	^16^O: 74.5%	^16^O: 88.9%
^12^C: 9.04%	^12^C: 9.04%	
^14^N: 3.21%	^14^N: 3.21%	
^31^P: 2.61%	^31^P: 2.61%	

In the present work, natural boron was considered and the density of normal and 100 ppm boron filled cases considered to be fixed at 1.27 g/cm^3^. For boron filled cytoplasm cases, it was assumed that the cell cytoplasm contained a user-defined amount of boron in ppm. In the present work, as an example, we considered 100 ppm of boron in cell cytoplasm and nucleus. In addition, it was assumed that the buffer medium above the cell array contained 100 ppm of boron. The changes in elemental composition and density can alter the efficiency of alpha particles generation, mainly as a result of nuclear reactions. Therefore, interested readers can modify the program to match their specific material composition and densities. Users can change these values according to their own needs using the present program. The entire program and its source code is available for free download from: https://figshare.com/articles/software/On_the_effectiveness_of_proton_boron_fusion_therapy_PBFT_at_cellular_level/19658166 and interested users can modify the material compositions and densities to match a specific case.

### Terminal-based PHITS editor

**Figure 4:**
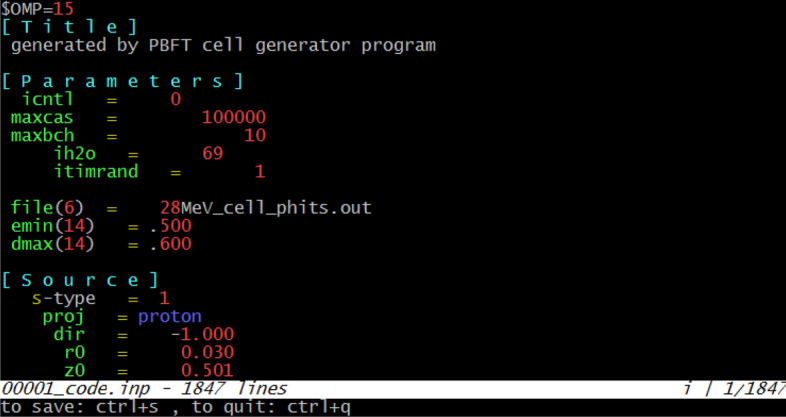
PHITS code editor program with PHITS syntax highlighting and line numbering in action.

It is important for users to have the ability to confidently edit their generated PHITS input script from the cell generator program. Normally, with large cell arrays, the number of cells and surfaces generated would be large. For example, for a 20 × 20 cell array (total of 400 cells) the generated PHITS input script would have about 1,812 lines of code. Most used text editors support syntax highlighting of most widely used programming languages such as; C/C++, Python, Fortran, Java etc. Some of the well-known and commonly used text editors are; gedit (https://wiki.gnome.org/Apps/Gedit), notepad++ (https://notepad-plus-plus.org/), atom (https://atom.io/), vi/vim (https://www.vim.org/), nano (https://www.nano-editor.org/), emacs (https://www.gnu.org/software/emacs/), etc. However, it is important to have a dedicated PHITS code editor with syntax highlighting and line numbering for ease of use in editing the generated input code. Currently, there are ~ 518 input commands that support syntax highlighting. The developed dedicated terminal-based code editor is coupled with the cell generator program and shown in action in Fig. 4.

### Program compilation and runtime

Two main compilers are used to generate executables of the present software, which are gfortran and gcc. The cell generator program uses gfortran compiler to compile the main FORTRAN90 program and all its subroutines (see Fig. 2), whereas the PHITS code editor program uses gcc compiler to compile the main C program and its linked header files. We have tested the program compilation with different versions of gcc package and this comparison is shown in Table 3. The compilation time highly depends on the computer system configurations, such as CPU clock speed, memory and type of storage drive (HDD or SSD).

**Table 3:** Compilation time versus different gcc package versions used to test the present program.

Gcc package version	Compile time (s)
5.3.0	2.524
9.4.0	0.664
11.2.0	0.332

The program runtime after the executable file was generated and the user input was read by the program was found to be less than 1 s. We have tested the present terminal-based cell generator program and the PHITS code editor on both Microsoft Windows and GNU/Linux platforms. However, since Microsoft Windows does not have a terminal as those present on GNU/Linux platforms, we have used Cygwin (https://www.cygwin.com/) and Windows Subsystem for Linux (WSL) (https://docs.microsoft.com/en-us/windows/wsl/) to test our programs. On both platforms no issues were found, and the program ran flawlessly. The Intra-Nuclear Cascade Liege (INCL) model was used to explain the nuclear reaction involved in the present work. The Intra-Nuclear Cascade of Liege (INCL) was implemented as the default model for simulating nuclear reactions induced by protons. The stopping powers for charged particles and nuclei were calculated using ATIMA code (https://web-docs.gsi.de/~weick/atima/) implemented in the PHITS MC package. In general, PHITS automatically calculates the stopping power of each material using ATIMA model.

### Computational scheme

In the present study, we have employed our developed cell generator program to study PBFT at the cellular level. The modelled cell array size was 20 × 20 having 400 cells each with the size of 30 × 30 × 30 µm^3^, given that the size of malignant HeLa cells was reported to be about 17–30 μm^24^. The size chosen here could appear larger than those usually encountered for normal cells. In relation, Shashni et al.^25^ revealed that the size parameters (e.g., inner diameter and width) for cancer cells were significantly greater than those for normal cells, and the inner diameters of many cancer cell lines were larger than 10 µm, which was uncommon for normal cells. The cell nucleus side length was set to be at 10 μm and placed at the center of each cell in the modelled array. Two different buffer medium thicknesses of 1000 and 5000 μm were considered and placed on top of the modelled cell array. In addition, three different incident proton beam energies of 25, 35 and 70 MeV were considered. In the present program, the incident proton beam was modelled as a disk source and the source radius was set to be 0.30 mm; this value was chosen to match half of the cell array side length and cover most cells. Some corner cells would not be irradiated directly by the incident proton beam, which could provide information on the transport of secondary radiations to non-irradiated cells. In addition, SOBP profile using an 80 MeV proton beam that was generated in the buffer medium layer. Similar monolayer cell array of 20 × 20 (having 400 cells) each with the size of 30 × 30 × 30 µm^3^, was placed at different positions under the buffer medium layer. Upon placing the cell array at different positions, essentially the thickness of buffer medium layer above the cell array was changed. These positions were 10^−4^, 4, 4.5 and 4.8 cm that represented the beam entrance, middle and distal end of the modelled SOBP profile. Two cases were considered, namely, (1) normal and (2) boron filled cytoplasm and nucleus. Similar to the previous discussions, 100 ppm of boron was also considered in the buffer medium layer, mainly to achieve a rather realistic setup. It needs to be noted that upon having 100 ppm of boron in the buffer medium, we have corrected exact thickness of buffer medium to match that of the buffer medium without boron; this is important to have a fair comparison between these two cases. The corresponding profile of generated SOBP within the buffer medium layer was obtained. The enhancement factors were calculated as an average ratio of the total dose alpha particle dose from boron filled and normal cytoplasm and nucleus cases. The cell generator program automatically generated tallies to score the output results from the PHITS Monte Carlo package. The tally parameters were automatically set by the program based on the modelled cell array size. The following tallies were considered in the program;

1. T-Gshow tally that produced graphical geometrical output of the modelled system.
2. T-Track tally that determined the fluence of all particles (proton, neutron and alpha) in the cell array.
3. T-Deposit tally that determined the energy deposition for each particle (proton, neutron and alpha) in the cell array domain.

The results obtained from the tallies were then analyzed with our previously developed PyBLD^26^ (http://www.rim.cyric.tohoku.ac.jp/software/pybld/pybld.html) program as follows;

1. Output simulation data as .out format.
2. PyBLD module used to convert .out data to .hdr and .img format by script_phitsout2ana.py script as;
  - python3 script_phitsout2ana.py deposit_alpha.out (this file was the output from the PHITS Monte Carlo package, which was used as input to the analysis program) d-a (this is the output data after the analysis).
  - Repeat step (2a) for all cases (i.e., incident proton energies and medium thicknesses) modelled in this work.
3. For all cases, the obtained 2D images were visualized by AMIDE^27^ (http://amide.sourceforge.net) software.
4. Superimpose the 2D images for proton, boron and alpha particles using AMIDE for visualization of tracks.
5. For the 1D profile data, save these into a CSV file and plot.

In the present study, the tracks of protons, neutrons, and alpha particles in the 20 × 20 array of cells with normal, boron filled, and random boron filled cytoplasm were determined for buffer medium thicknesses of 1000 and 5000 μm, and for incident proton energies of 25, 35 and 70 MeV. In addition, the axial doses from alpha particles within the cell array were obtained for all three cytoplasm scenarios, and for the two buffer medium thicknesses and the three incident proton energies. The average computational time taken for the cases shown in this section was found to be about 16.4 h. The computations were performed on dual Xeon E5-2630 v3 2.40 GHz and Xeon Silver 4210 2.20 GHz using 8 to 30 cores. The option for time dependent initial random number seeds was implemented and enabled; this ensured changes in the initial random number seeds in all modelled cases. Considering the fact that the monolayer cell array is rather a small target and random initial seed was implemented, the computations were performed with different number of batches (i.e., *maxbch*) and the average results and its respective statistical uncertainties were obtained and shown here; this is mainly to reduce statistical uncertainties and achieve a rather fair comparison between the results of normal and boron filled cases. Users can simply set different batch runs by setting *maxbch* in the present program as shown in Table 1.

## Results and discussion

### Particle tracks in cell array

**Figure 5:**
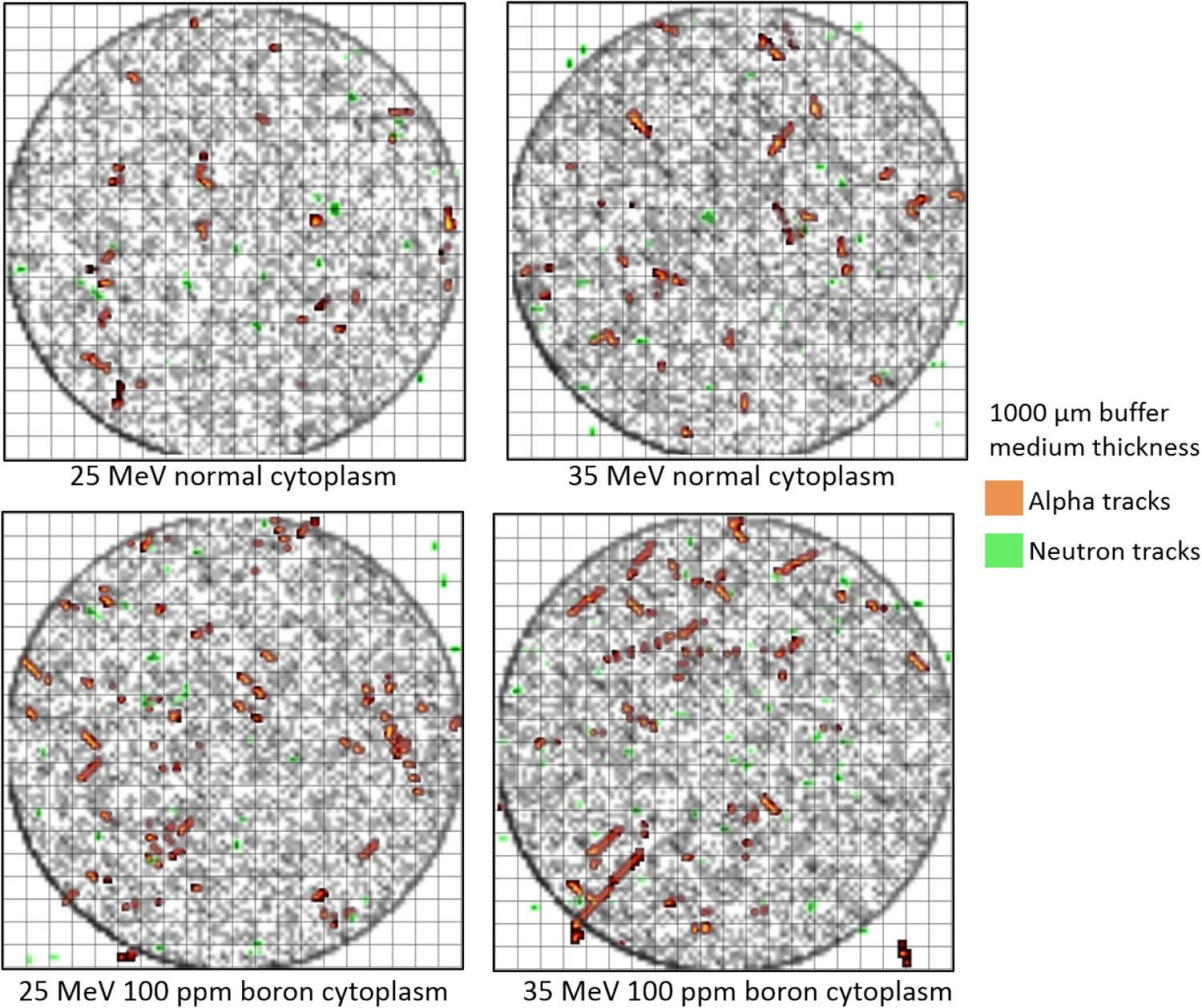
The tracks of alpha particles, neutrons and incident protons (shown in black) generated as a result of 25 and 35 MeV irradiation of cell array with buffer medium thickness of 1000 μm.

**Figure 6:**
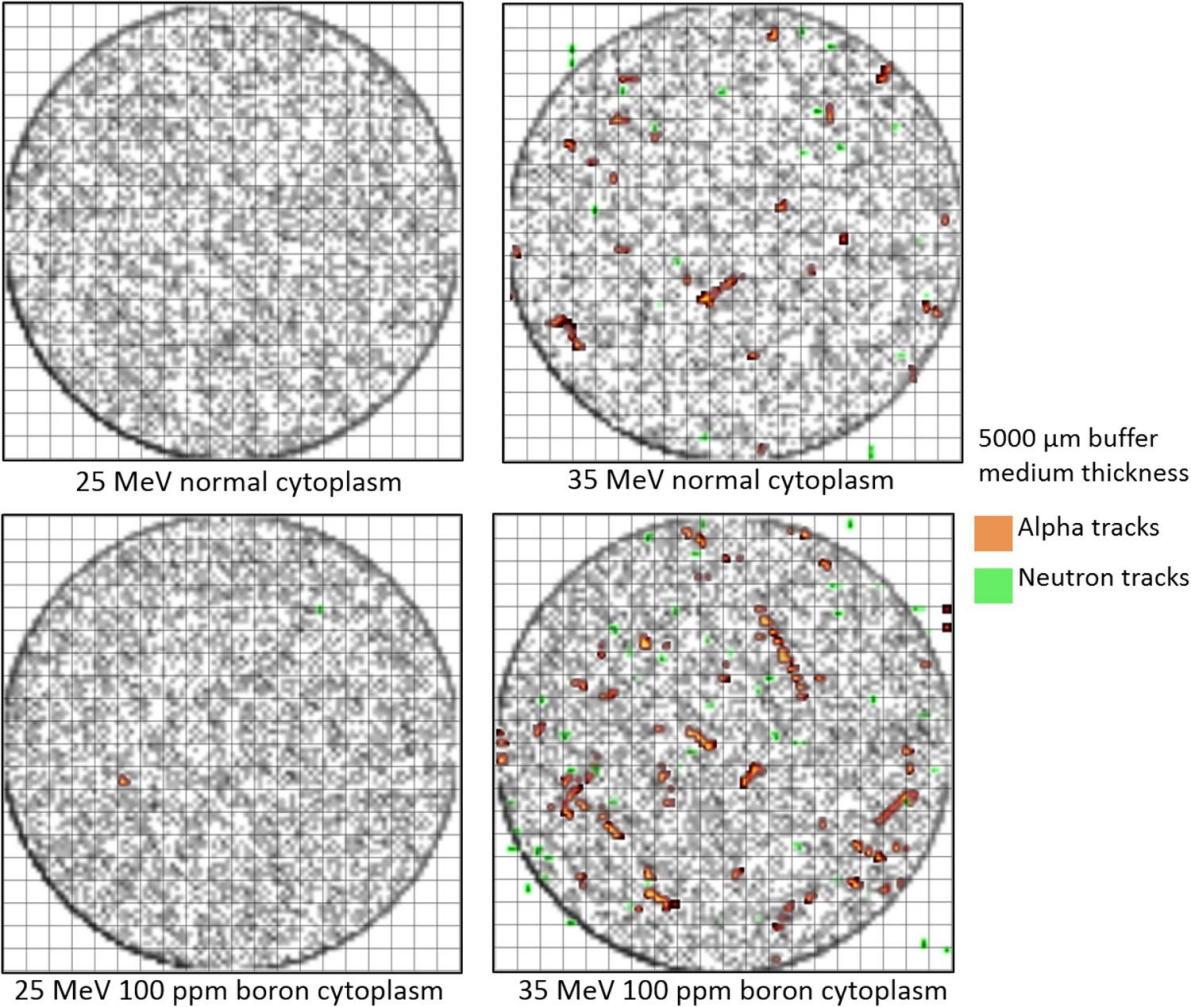
The tracks of alpha particles, neutrons and incident protons (shown in black) generated as a result of 25 and 35 MeV irradiation of cell array with buffer medium thickness of 5000 μm.

The results of the modelled cell array with 400 cells were obtained and then analyzed to produce 2-dimensional images of alpha particle, neutron, and proton tracks in the modelled cell array. The results from this analysis for buffer medium thicknesses of 1000 and 5000 μm above the cell layer are shown in Figs. 5 and 6, respectively.

In Figs. 5 and 6, the employed proton disk source was clearly discernable seen (i.e., the black circle and tracks). Although most alpha particles and neutrons generated within the irradiation zone (i.e., within the black circle), some of the generated alpha particle and neutron tracks did go beyond the irradiation zone. It was noticed that alpha particle tracks increased when the cytoplasm and nucleus of cells were filled with 100 ppm of boron (note: see Table 4 for quantitative comparison for hit counts in the cell array from alpha particles) which was expected as production of alpha particles upon interaction between proton and boron was the mechanism underlying PBFT. Interestingly, for the case of normal cytoplasm (i.e., no boron present in the cells), alpha particles would still be produced, which was attributed to nuclear reactions induced by proton interaction with ^16^O, ^12^C and ^14^N nuclei. For example, one of the dominant alpha producing reactions that has a relatively low threshold of 5.66 MeV is the ^16^O(p,α)^13^N nuclear reaction^28^. We previously studied the use of this reaction and the produced ^13^N radioisotopes for proton range monitoring and verification^29–31^. These alpha-particle producing nuclear reactions can be found from the Experimental Nuclear Reaction Data (EXFOR) (https://www-nds.iaea.org/exfor/) database. Our previous study revealed that upon proton interaction mainly with ^16^O and ^14^N nuclei that are alpha-particle producing reactions, ^13^N (as a result of ^16^O(p,α)^13^N nuclear reaction) and ^11^C (as a result of ^14^N(p,α)^11^C nuclear reaction^29–32^) radioisotopes would be produced. In the case of normal cytoplasm, we also noticed the production of these radioisotopes in the buffer medium and the cell layer, which suggested that alpha particles were produced in these domains as a result of nuclear interaction with protons in absence of boron. To further confirm this, we developed a hypothetical cell array that only contained hydrogen (^1^H), from which no alpha particles were produced upon proton irradiation. It needs to be noted that, for 100 ppm of boron in cell cytoplasm and nucleus, the production of alpha particles as a result of proton interaction with ^16^O nuclei is another significant mechanism for production of alpha particles.

**Table 4:** Hit counts in the cell array from alpha particles for incident proton energies of 25 and 35 MeV with buffer medium thickness of 1000 µm and 5000 μm, for normal and boron filled cytoplasm and nucleus.

Case (incident proton energy/buffer medium)	Normal (nucleus)	Normal (cytoplasm)	Boron filled (nucleus)	Boron filled (cytoplasm)
25 MeV/1000 μm	(26,47,68)	(111,195,279)	(43,63,83)	(170,247,324)
25 MeV/5000 μm	(0,0,0)	(0,1,2)	(0,1,3)	(0,2,7)
35 MeV/1000 μm	(26,56,86)	(94,190,286)	(41,68,95)	(168,249,330)
35 MeV/5000 μm	(27,52,77)	(115,226,337)	(34,59,84)	(141,241,341)

The results present (lower bound, average, upper bound) values of hit counts obtained from three runs.

### Axial energy deposition of alpha particles in cell layer

**Figure 7:**
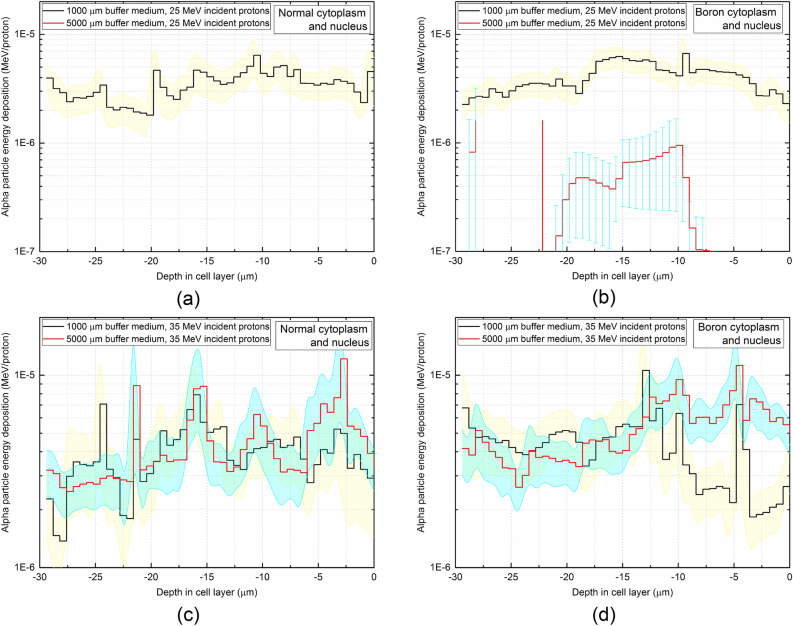
Axial energy deposition of alpha particles in the cell layer impinged by 25 MeV protons having (**a**) normal, (**b**) boron filled cytoplasm and nucleus, and impinged by 35 MeV protons having (**c**) normal, (**d**) boron filled cytoplasm and nucleus. The yellow and blue highlight represent the statistical uncertainties associated with the Monte Carlo computations for 1000 and 5000 μm buffer medium thickness, respectively.

The axial energy deposition in the cell layers from incident protons with energies of 25 and 35 MeV for normal and boron filled cytoplasm and nucleus were obtained and shown in Fig. 7. The cell array was generated along negative z-axis, and the depth in the cell layer starts from 0 (i.e., top surface of the cell array underneath the buffer medium) to − 30 μm (i.e., bottom surface of the cell array). For incident protons with the energy of 25 MeV, the results for normal cytoplasm (see Fig. 7a) and boron filled cytoplasm and nucleus (see Fig. 7b) showed that alpha particle energy deposition within the cell array increased in the presence of boron (see Fig. 10 for total energy deposition of alpha particles in the modelled cell array), which was explained by the proton boron nuclear reaction leading to generation of alpha particles which in turn deposited more energy in the cell array. Interestingly, no alpha particle energy deposition was detected for 5000 μm buffer medium for normal cytoplasm cells irradiated with 25 MeV incident proton beam; this is due to the fact that energy of protons in the cell layer decreased below the threshold value for ^16^O(p,α)^13^N nuclear reaction. However, in presence of boron filled cytoplasm and nucleus some energy deposition from alpha particles were detected for 5000 μm buffer medium (see Fig. 7b). The production of these alpha particles was due to the PBFT reaction, which has lower probability at very low energies and presents a low efficiency of production at lower concentration of boron. From the cross-section data of the PBFT process^33^, the efficiency of proton boron nuclear reaction increased with decreasing proton energy, up to reaching a maximum point and then starts to decrease for lower energy of protons.

However, the results showed that for the incident proton energy of 35 MeV, a thicker buffer medium thickness led to a larger deposition of alpha particle energy in the cell layer below, which was mainly due to the energy reduction of protons that falls within the optimal energy for the PBFT reaction^14,33^. From the results shown in Fig. 7d, it can be seen that thicker buffer medium would lead to increase in the energy deposition from alpha particles mainly 15 μm away from the proton beam entrance. For relatively higher incident proton energies, most alpha particles would be produced in the buffer medium layer (since the modelled medium layer was much thicker than the cell thickness), for both normal and boron filled cytoplasm and nucleus. Some of these generated alpha particles in the buffer medium can travel and interact with the cell layer underneath. However, considering the fact that alpha particles are densely ionizing in nature and possess relatively short ranges in mediums, propagation of alpha particles from the buffer medium layer into the underneath cell layer would be strongly dependent on the thickness of the buffer medium (i.e., the distance that needs to be traveled to reach the underneath cell array).

**Figure 8:**
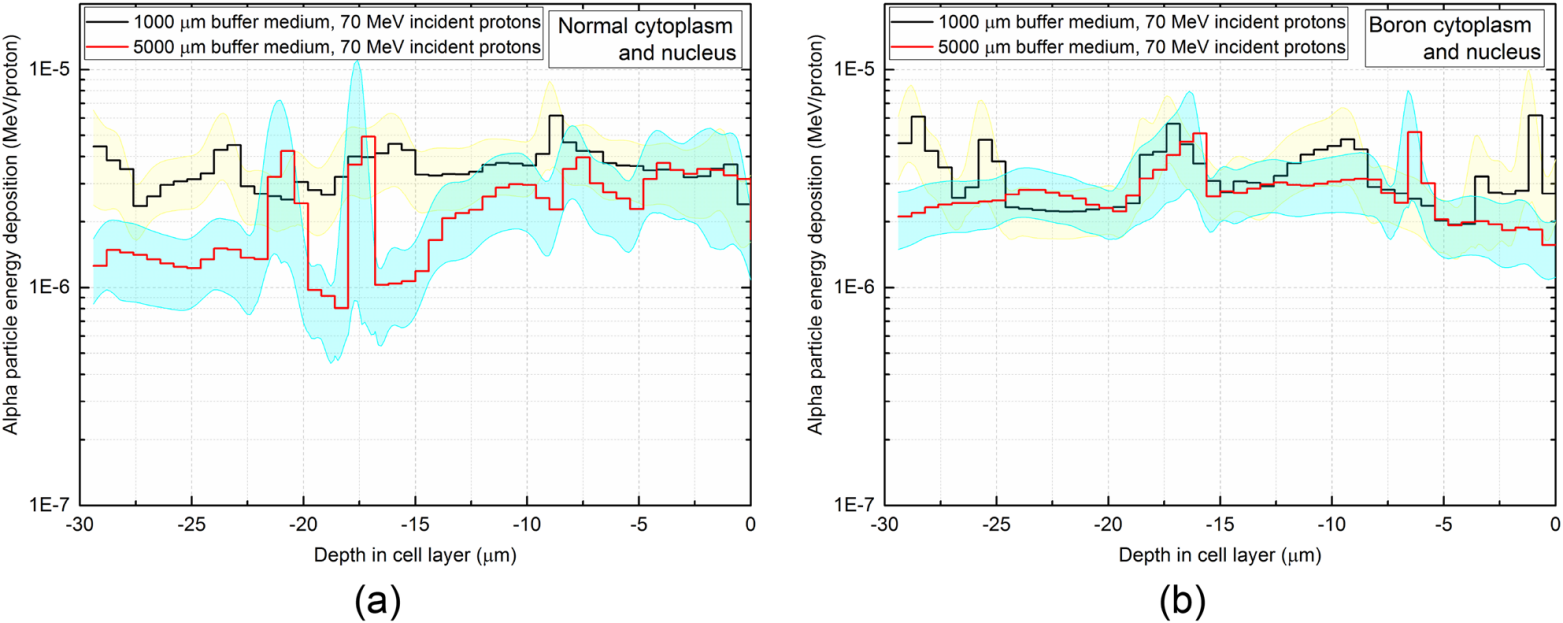
Axial energy deposition of alpha particles in the cell layer impinged by 70 MeV protons having (**a**) normal, (**b**) boron filled cytoplasm and nucleus. The yellow and blue highlight represent the statistical uncertainties associated with the Monte Carlo computations for 1000 and 5000 μm buffer medium thickness, respectively.

The results for the incident proton energy of 35 MeV for the boron filled cytoplasm shown in Fig. 7d showed that the energy deposition of alpha particles in the cell array was not significantly reduced for a thicker buffer medium layer when compared to the case of the normal cytoplasm. With the presence of boron, alpha particles would be produced within the cell layer, which could offset the energy deposition in the cell layer below the thicker buffer medium. We confirmed this by further increasing the incident energy of protons to 70 MeV and calculating the energy deposition of alpha particles within the cell layer; these results are shown in Fig. 8.

Figure 8 shows that for the boron filled cytoplasm and nucleus the energy deposition of alpha particles in the cell array for the thicker buffer medium thickness (i.e., 5000 μm buffer medium) tend to be close to the case for the thinner buffer medium thickness (i.e., 1000 μm buffer medium). This difference for the incident proton energy of 70 MeV is less significant than that for 35 MeV due to more alpha particles produced in the buffer medium by the ^16^O(p,α)^13^N nuclear reaction, which has a relatively low energy threshold. For higher incident energy of proton, the presence of boron in the cell layer cannot effectively offset the energy deposition in the cell layer; this is mainly due to the fact that the incident energy of protons is rather too high for proton boron fusion reaction to have significant enhancement. Ideally, thicker buffer medium layers could contribute to the enhancement of proton boron fusion reaction. However, for very high energy incident proton energies, the 5000 μm medium layer is incapable of reducing the proton energy effectively.. Interestingly, for the incident proton energy of 70 MeV, the boron filled cytoplasm and nucleus showed no significant enhancements in deposition of alpha particle energy when compared to the normal cytoplasm, as the dominant alpha particle producing channel is ^16^O(p,α)^13^N nuclear reaction. It is important to note that ^16^O(p,α)^13^N nuclear reaction plays an important role in production of alpha particles. The fluctuations and spikes in the alpha particle energy deposition versus depth in the cell layer is mainly due to the asymmetric production and interaction of alpha particles in the cell array and highly localized energy deposition at certain locations, respectively. This asymmetry can be seen from the track visualizations shown in Figs. 5 and 6, as the alpha particles in the cell array dispersed in various directions and in turn lead to diverse alpha particle density at different locations in the cell array.

### Random boron distribution and total alpha particle energy deposition

**Figure 9:**
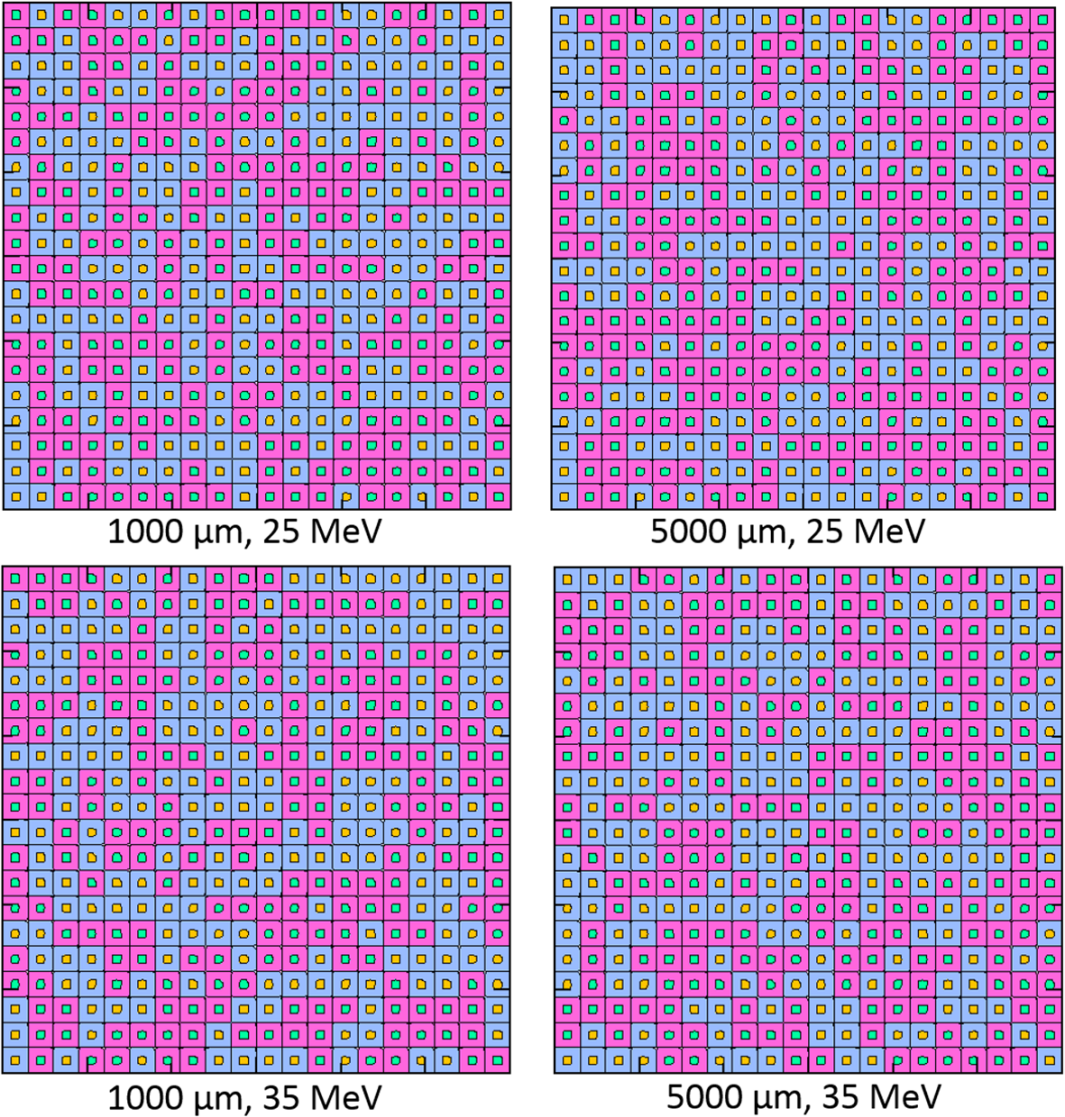
Examples of random boron distribution in the generated cell array below 1000 and 5000 µm buffer media irradiated by protons with incident energies of 25 and 35 MeV. (Blue and pink represent normal and boron filled cytoplasm, respectively. Orange and green represent normal and boron filled cell nucleus, respectively).

For the random boron distribution case, the cell generator program assigns random boron filling positions on the modelled cell array. The random seed changes for different simulation cases and examples of the generated random arrays are shown in Fig. 9. It is rather tedious to visualize alpha particle tracks for random boron distribution cases, since the position of boron filled cytoplasm must be superimposed onto the tracks. Therefore, for simplicity we have not performed track visualization for the random boron distribution cases. However, the numerical results from the random boron distribution cases were used for the comparison. Only those cells with boron filled cytoplasm would have boron filled nucleus.

**Figure 10:**
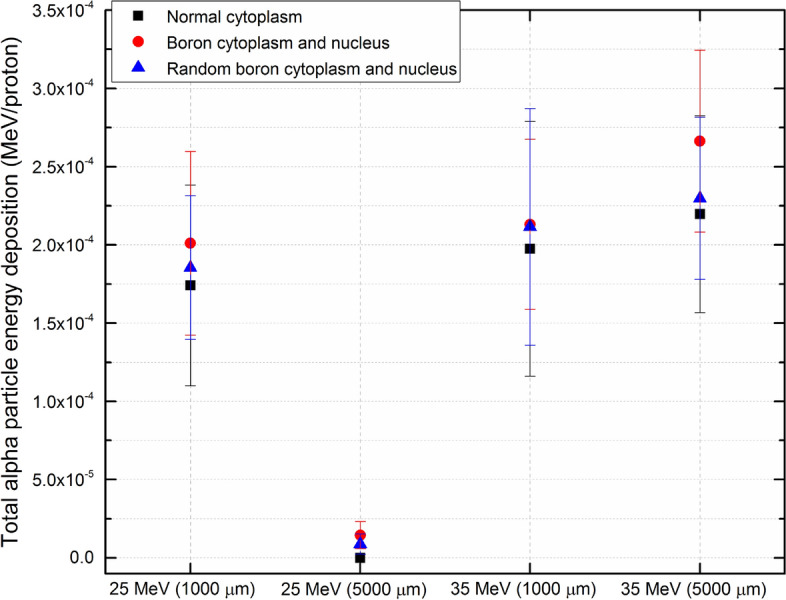
The total alpha particle energy deposition for different incident proton energies and for different buffer medium thicknesses, for boron and random boron filled cytoplasm and nucleus.

In order to better compare the effectiveness of alpha particle energy deposition for normal and boron filled cytoplasm as shown in Fig. 7, the total energy deposition in the cell layer was calculated. Similarly, the total energy deposition in the cell layer for random boron distribution cases as shown in Fig. 9 was also computed. The total alpha particle energy deposition for different incident proton energies and for different buffer medium thicknesses are shown in Fig. 10.

From Fig. 10, boron filled cytoplasm and nucleus were found to have higher total alpha particle energy deposition in the modelled cell array. This is mainly due to the fact that PBFT and ^16^O(p,α)^13^N nuclear reaction both have dominant contribution in boron filled cytoplasm and nucleus cases, which leads to higher average alpha particle energy deposition in the modelled cell array. The random boron filled cytoplasm and nucleus case were found to have the second highest average alpha particle energy deposition in the modelled cell array, mainly due to the fact that the contribution from PBFT reaction would be lowered as a result of random distribution of boron. In fact, the total alpha particle energy depositions are arguably highly case dependent, depending on the incident proton energy, size of the source and cell array, buffer medium thickness, the concentration and distribution of boron in the cell array. It is not feasible to report total alpha particle energy depositions for all possible cases, and the solution to this issue would be therefore to develop software tools as those reported in the present work, so that the users can compute the average alpha particle energy depositions for their specific irradiation scenarios. All the software tools, including their source code, and the results shown in the present work can be found from: https://figshare.com/articles/software/On_the_effectiveness_of_proton_boron_fusion_therapy_PBFT_at_cellular_level/19658166.

### Fraction of energy deposition

**Figure 11:**
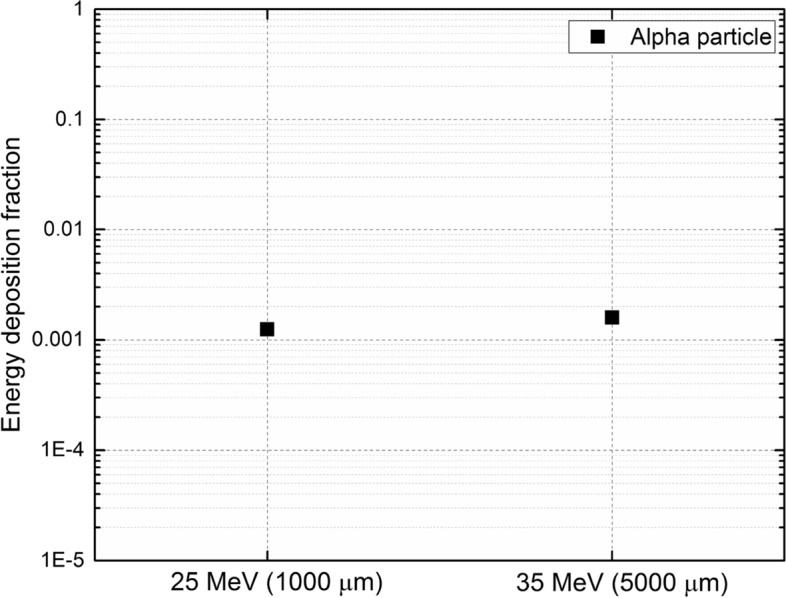
Fraction of energy deposition from alpha particles for (1) incident proton energy of 25 MeV with buffer medium thickness of 1000 µm, and (2) incident proton energy of 35 MeV with buffer medium thickness 5000 μm, both having boron filled cytoplasm and nucleus.

The total proton and alpha particle energy deposition in the cell array were used to determine the fraction of energy deposition from primary incident protons. These results were directly obtained from PHITS and are normalized per primary source particle, therefore explicit determination of particle flux would not be required^34,35^. The fraction of energy deposition from alpha particles for the two cases of 25 and 35 MeV irradiating the modelled cell array with 1000 and 5000 μm buffer medium thickness, respectively, are shown in Fig. 11.

Figure 11 shows that for boron filled cytoplasm and nucleus, the energy deposition fraction of alpha particles was attributed to the dominance of production of alpha particles in the cells and the subsequent deposition of their energy within the cell array.

The fraction of deposited energy from alpha particles compared to incident protons is not entirely negligible, despite the previously reported results^16^. It needs to be noted that the definition of dose is rather ambiguous here, as the mass of the target or more specifically the location at which energy has been deposited can vary significantly. The effectiveness of PBFT or generally any radiation therapy mode could only be assessed fairly by investigating the interaction events and consecutively energy depositions at the cellular level since killing of cancer cells is the ultimate goal in radiation therapy. In addition, different types of ionizing radiation, even those within the same general classification (i.e., directly or indirectly ionizing radiation), induce different types of damage to the cells and DNA molecules^36,37^. We strongly believe that simply comparing dose components would not be an accurate way to judge the effectiveness of a treatment modality for a large sized and oversimplified target phantom as recently reported in Refs.^16,38,39^. We believe that the relative biological effectiveness (RBE) weighted doses by attributing maximum potential RBEs to the dose of alphas would be incapable of explaining the significance of the biological effect observed in PBFT, therefore more investigations would be needed in this regard.

### The Spread-Out Bragg peak (SOBP)

**Figure 12:**
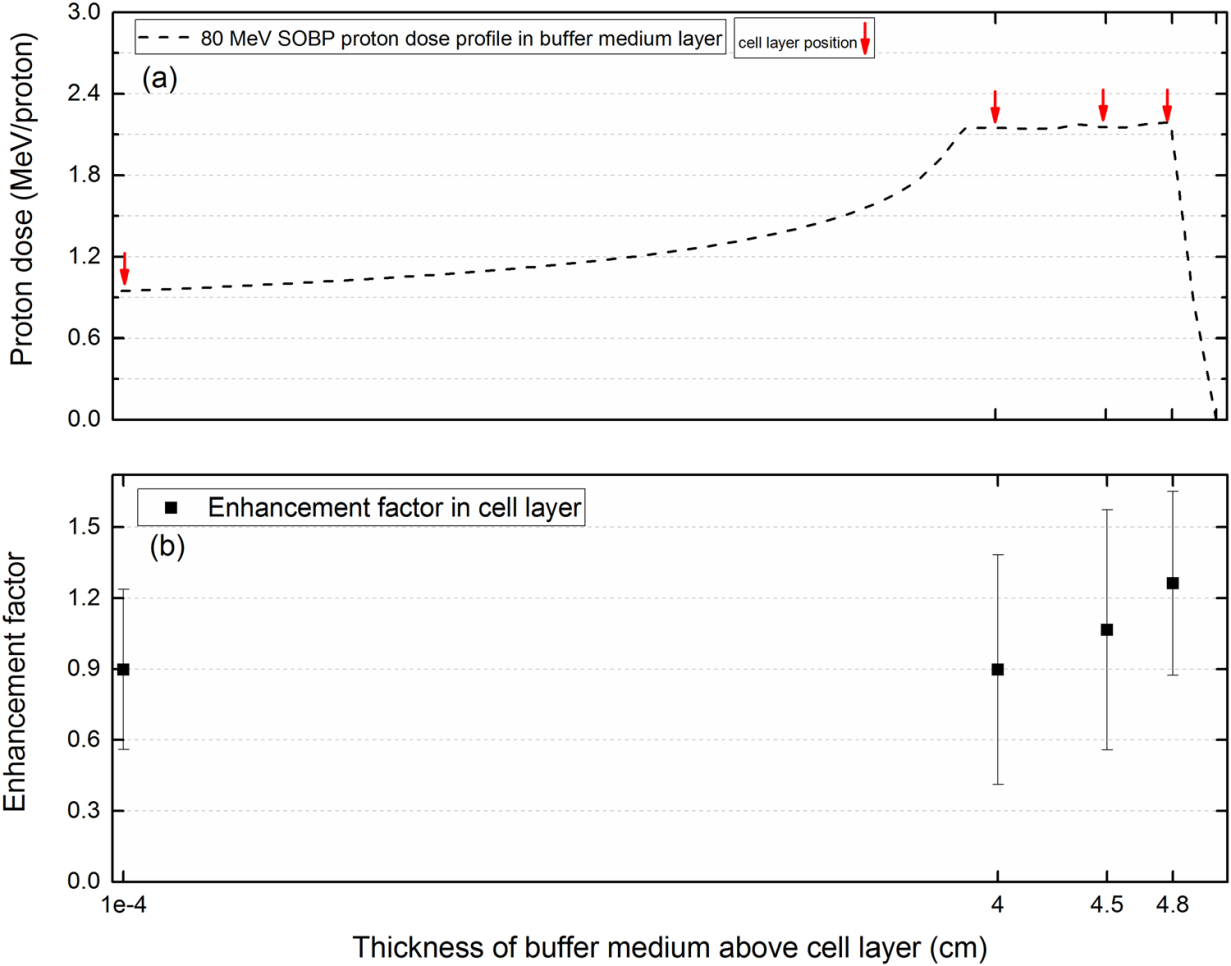
The (**a**) SOBP profile in the buffer medium layer and (**b**) enhancement factors obtained in the monolayer cell array placed underneath of the buffer medium layer. (Red arrows represent the cell array position).

Figure 12a shows the modelled obtained SOBP profile in the buffer medium layer with cell layer position marked by arrows. The energy of protons that interact with the underneath cell array decreases when the cell array position gets closer to the distal end of the SOBP (i.e., 4.8 cm). The obtained enhancement factors that were obtained in the monolayer cell array at different thicknesses of buffer medium above the monolayer cell array are shown in Fig. 12b.

From the obtained enhancement factors that were shown in Fig. 12b, the enhancement factors start to increase as getting close to the distal end of the SOBP. The enhancement factor at the entrance region (i.e., 10^–4^ cm) found to be close to 1, which implies the fact that at the entrance region in which the energy of incident protons are highest, addition of 100 ppm boron would essentially have no effect on the total alpha particle dose deposited in the monolayer cell array. The main reason behind this, is the ineffectiveness of PBFT reaction at high proton energies, which essentially leads to no significant enhancement of alpha particle dose in the monolayer cell array.

Further increasing the thickness of the buffer medium layer, the total alpha particle dose deposition in the cell array underneath of buffer medium starts to increase, which shows the enhancement of alpha particle dose deposition in presence of boron. The main reason for this enhancement would the PBFT reaction that has higher effectiveness at lower proton energies (i.e., closer to the distal end of SOBP) that in turn leads to production of more alpha particles.

The present results and the enhancement factors that were introduced, showed the importance of alpha particle dose enhancement in cellular constituents (i.e., cytoplasm and nucleus) in presence and absence of boron. For different experiment situations, important parameters such as cell array dimensions, number of cell layers, location of cells within the array, concentration of boron, incident proton energy etc. can vary significantly. Therefore, it is strongly recommended that experimental investigators report all these parameters that would be needed in the model developed in the present work.

### Alpha particle hit counts

In order to fairly compare the effectiveness of PBFT, it is pertinent to compare the alpha particle hits within the cytoplasm and nucleus of the cell array, since the hits/tracks of a densely ionizing radiation such as alpha particle would be an important characteristic to assess the effectiveness of cell killing. For this comparison, we have determined the hits of alpha particles in cell cytoplasm and nucleus for incident proton energies of 25 and 35 MeV, and for buffer medium thicknesses of 1000 and 5000 μm, in cells with normal and boron filled cytoplasm and nucleus. The comparison was carried out for normal and boron filled cytoplasm and nucleus cases. The hits counts were obtained using a custom-made Python script named “count_nucleus.py” bundled with the PyBLD^26^ (http://www.rim.cyric.tohoku.ac.jp/software/pybld/pybld.html) program. The script and other tools used in the present work can be downloaded from: https://figshare.com/articles/software/On_the_effectiveness_of_proton_boron_fusion_therapy_PBFT_at_cellular_level/19658166. The script reads the hits/track output file from PHITS Monte Carlo package and locations of cell nucleus and cytoplasm and reports the hit counts for alpha particles. The results are summarized in Table 4.

From Table 4, one can see that the number of alpha particles hits in the cell cytoplasm and nucleus increases in the presence of boron. The increases in the alpha particle hits in cell cytoplasm and nucleus were found to be rather subtle, which was due to the presence of 100 ppm of boron in the boron filled cytoplasm and nucleus cases. As mentioned previously, the production of alpha particles as a result of proton interaction with ^16^O nuclei was another significant mechanism for production of alpha particles. This demonstrates that cells with boron filled cytoplasm and nucleus would get more alpha particle hits. The present track analysis tool and example results would be a useful for fair assessment of PBFT for different user-defined cases such as, different cell array sizes, incident proton energies, boron distribution and buffer medium thicknesses.

## Conclusions

In the present work, a terminal-based cell generator program was developed that generated PHITS input script for PBFT applications in large cell arrays. The program enabled users to generate cell arrays with normal, boron filled as well as random boron filled cytoplasm and nucleus with user-defined boron concentration in ppm. In addition to the cell array generator, a dedicated terminal-based PHITS code editor was developed that enabled syntax highlighting and made the code editing task easier for users, considering that for large cell arrays the input script would be rather lengthy.

Radiation track visualizations in the cell array layer were enabled and the required tools were provided to generate 2D track visualization. The variation of energy deposition by alpha particles in the cell array versus the depth within the cell layer was also determined. The effects of incident proton energy and buffer medium layer thickness were also studied. To quantitatively represent the effectiveness of alpha particle energy deposition for different scenarios, the average energy deposition by alpha particles were determined. Furthermore, the fractions of energy deposition from alpha particles over energy deposition from incident protons were also determined. Lastly, the hit counts were determined for alpha particles in cell cytoplasm and nucleus. A number of important observations were made in the present study, namely,

1. Alpha particle and neutron tracks move beyond the source irradiation zone.
2. In the absence of boron, alpha particles are produced in the nuclear reaction between protons and ^16^O, ^12^C and ^14^N nuclei.
3. The alpha particle energy deposition within the cell array generally increases with the buffer medium thickness; however, for higher incident proton energy, a thicker buffer medium leads to no significant changes of alpha energy deposition in the cell layer.
4. For relatively higher incident proton energies, most alpha particles are produced in the buffer medium layer for both normal and boron filled cytoplasm and nucleus.
5. Boron filled cases tend to provide higher alpha particle energy deposition in the cell array.
6. The total energy deposition of alpha particles is in fact highly case dependent, mainly depending on the incident proton energy, source size, cell array size, buffer medium thickness, concentration and distribution of boron in the cell array.
7. The fraction of deposited energy from alpha particles compared to that from incident protons is not entirely negligible.
8. The hits count of alpha particles in cell cytoplasm and nucleus increase in the presence of boron.
9. Simply comparing dose components would not be an accurate way to judge the effectiveness of a treatment modality.

We believe the obtained results and the developed tools would be useful for future development of PBFT and for fairly assessing the effectiveness of this treatment modality for different scenarios. In future works, we aim to develop cell models with more complex geometrical details. All data, codes and results in the present work are freely available for download from: https://figshare.com/articles/software/On_the_effectiveness_of_proton_boron_fusion_therapy_PBFT_at_cellular_level/19658166.

## Data Availability

All data files and the open-source program presented in this work are available from Figshare database (https://figshare.com/articles/software/On_the_effectiveness_of_proton_boron_fusion_therapy_PBFT_at_cellular_level/19658166).

## References

[1] Hall E, Wu CS (2003). Radiation-induced second cancers: The impact of 3D-CRT and IMRT. Int. J. Radiat. Oncol. Biol. Phys..

[2] Howell RM, Hertel NE, Wang Z, Hutchinson J, Fullerton GD (2006). Calculation of effective dose from measurements of secondary neutron spectra and scattered photon dose from dynamic MLC IMRT for 6 MV, 15 MV, and 18 MV beam energies. Med. Phys..

[3] Xu XG, Bednarz B, Paganetti H (2008). A review of clinical data and radiation dosimetry methods on secondary cancers from external beam radiation treatments. Phys. Med. Biol..

[4] Shahmohammadi Beni M, Ng CYP, Krstic D, Nikezic D, Yu KN (2017). Conversion coefficients for determination of dispersed photon dose during radiotherapy: NRUrad input code for MCNP. PLoS ONE.

[5] Shahmohammadi Beni M, Krstic D, Nikezic D, Yu KN (2021). A comparative study on dispersed doses during photon and proton radiation therapy in pediatric applications. PLoS ONE.

[6] Shahmohammadi Beni M, Watabe H, Krstic D, Nikezic D, Yu KN (2021). MCHP (Monte Carlo+ Human Phantom): Platform to facilitate teaching nuclear radiation physics. PLoS ONE.

[7] Yoon DK, Jung JY, Suh TS (2014). Application of proton boron fusion reaction to radiation therapy: A Monte Carlo simulation study. Appl. Phys. Lett..

[8] Amaldi U, Kraft G (2005). Radiotherapy with beams of carbon ions. Rep. Prog. Phys..

[9] Ward JF (1994). The complexity of DNA damage: Relevance to biological consequences. Int. J. Radiat. Biol..

[10] Goodhead DT (1994). Initial events in the cellular effects of ionizing radiations: Clustered damage in DNA. Int. J. Radiat. Biol..

[11] Hada M, Sutherland BM (2006). Spectrum of complex DNA damages depends on the incident radiation. Radiat. Res..

[12] Suzuki M, Kase Y, Yamaguchi H, Kanai T, Ando K (2000). Relative biological effectiveness for cell-killing effect on various human cell lines irradiated with heavy-ion medical accelerator in Chiba (HIMAC) carbon-ion beams. Int. J. Radiat. Oncol. Biol. Phys..

[13] Facoetti A (2015). In vivo radiobiological assessment of the new clinical carbon ion beams at CNAO. Radiat. Prot. Dosim..

[14] Cirrone GAP (2018). First experimental proof of Proton Boron Capture Therapy (PBCT) to enhance protontherapy effectiveness. Sci. Rep..

[15] Yoon DK (2019). Application of proton boron fusion to proton therapy: Experimental verification to detect the alpha particles. Appl. Phys. Lett..

[16] Chiniforoush TA, Hadadi A, Kasesaz Y, Sardjono Y (2021). Evaluation of effectiveness of equivalent dose during proton boron fusion therapy (PBFT) for brain cancer: A Monte Carlo study. Appl. Radiat. Isot..

[17] Shahmohammadi Beni M, Krstic D, Nikezic D, Yu KN (2016). A calibration method for realistic neutron dosimetry in radiobiological experiments assisted by MCNP simulation. J. Radiat. Res..

[18] Shahmohammadi Beni M, Krstic D, Nikezic D, Yu KN (2017). Realistic dosimetry for studies on biological responses to X-rays and γ-rays. J. Radiat. Res..

[19] Hsiao Y, Stewart RD (2007). Monte Carlo simulation of DNA damage induction by x-rays and selected radioisotopes. Phys. Med. Biol..

[20] Barberet P (2012). Monte-Carlo dosimetry on a realistic cell monolayer geometry exposed to alpha particles. Phys. Med. Biol..

[21] Neshasteh-Riz A, Koosha F, Mohsenifar A, Mahdavi SR (2012). DNA damage induced in glioblastoma cells by I-131: A comparison between experimental data and Monte Carlo simulation. Cell J..

[22] Clarke SD, Jevremovic T (2005). MCNP5 evaluation of dose dissipation in tissue-like media exposed to low-energy monoenergetic X-ray microbeam. Radiat. Environ. Biophys..

[23] Shahmohammadi Beni M, Krstic D, Nikezic D, Yu KN (2019). Medium-thickness-dependent proton dosimetry for radiobiological experiments. Sci. Rep..

[24] Nath B (2018). Understanding flow dynamics, viability and metastatic potency of cervical cancer (HeLa) cells through constricted microchannel. Sci. Rep..

[25] Shashni B (2018). Size-based differentiation of cancer and normal cells by a particle size analyzer assisted by a cell-recognition PC software. Biol. Pharm. Bull..

[26] Carson, R. E., Huang, S. C. & Phelps, M. E. BLD: A Software system for physiological data handling and model analysis. *Proc. Annu. Symp. Comput. Appl. Med. Care* 562–565 (1981).

[27] Loening AM, Gambhir SS (2003). AMIDE: A free software tool for multimodality medical image analysis. Mol. Imaging.

[28] Cho J (2017). Feasibility study of using fall-off gradients of early and late PET scans for proton range verification. Med. Phys..

[29] Islam MR (2022). Proton range monitoring using 13N peak for proton therapy applications. PLoS ONE.

[30] Islam MR (2022). An analysis scheme for 3d visualization of positron emitting radioisotopes using positron emission mammography system. Appl. Sci..

[31] Shahmohammadi Beni M, Yu KN, Islam MR, Watabe H (2022). Development of PHITS graphical user interface for simulation of positron emitting radioisotopes production in common biological materials during proton therapy. J. Radiat. Res..

[32] Beebe-Wang J, Vaska P, Dilmanian FA, Peggs SG, Schlyer DJ (2003). Simulation of proton therapy treatment verification via PET imaging of induced positron-emitters. IEEE Nucl. Sci. Symp. Conf. Rec..

[33] Geser FA, Valente M (2020). A theoretical model for the cross section of the proton-boron fusion nuclear reaction. Radiat. Phys. Chem..

[34] Shahmohammadi Beni M, Krstic D, Nikezic D, Yu KN (2018). Monte Carlo studies on photon interactions in radiobiological experiments. PLoS ONE.

[35] Shahmohammadi Beni M, Hau TC, Krstic D, Nikezic D, Yu KN (2017). Monte Carlo studies on neutron interactions in radiobiological experiments. PLoS ONE.

[36] Sutherland BM, Bennett PV, Sidorkina O, Laval J (2000). Clustered DNA damages induced in isolated DNA and in human cells by low doses of ionizing radiation. Proc. Natl. Acad. Sci. U.S.A..

[37] Friedland W, Kundrát P (2013). Track structure based modelling of chromosome aberrations after photon and alpha-particle irradiation. Mutat. Res. Genet. Toxicol. Environ. Mutagen..

[38] Meyer HJ, Titt U, Mohan R (2022). Monte Carlo study of the mechanism of proton–boron fusion therapy. Med. Phys..

[39] Khaledi N, Wang X, Hosseinabadi RB, Samiei F (2021). Is the proton–boron fusion therapy effective?. J. Radiother. Pract..

